# The Comparative Evaluation of the Antimicrobial Effect of Propolis with Chlorhexidine against Oral Pathogens: An In Vitro Study

**DOI:** 10.1155/2016/3627463

**Published:** 2016-02-02

**Authors:** A. Eralp Akca, Gülçin Akca, Fulya Toksoy Topçu, Enis Macit, Levent Pikdöken, I. Şerif Özgen

**Affiliations:** ^1^Faculty of Dentistry, Department of Periodontology, Istanbul Kemerburgaz University, Mahmutbey, 34217 Istanbul, Turkey; ^2^Gazi University Faculty of Dentistry, Department of Medical Microbiology, Emek, 06510 Ankara, Turkey; ^3^Gulhane Military Medical Academy, Center for Dental Sciences, Department of Restorative Dentistry, Etlik, 06010 Ankara, Turkey; ^4^Gulhane Military Medical Academy, Department of Analytical Toxicology, Etlik, 06010 Ankara, Turkey; ^5^Gulhane Military Medical Academy, Haydarpasa Training Hospital, Department of Dentistry, Section of Periodontology, Haydarpasa, 34668 Istanbul, Turkey; ^6^Faculty of Dentistry, Department of Oral Surgery, Istanbul Kemerburgaz University, Mahmutbey, 34217 Istanbul, Turkey

## Abstract

This study aimed to compare the antimicrobial effectiveness of ethanolic extract of propolis (EEP) to chlorhexidine gluconate (CHX) on planktonic* Streptococcus mutans*,* Streptococcus sobrinus*,* Lactobacillus acidophilus*,* Lactobacillus salivarius* subsp.* salivarius*,* Aggregatibacter actinomycetemcomitans*,* Prevotella intermedia*,* Porphyromonas gingivalis*,* Staphylococcus aureus*,* Enterococcus faecalis*,* Actinomyces israelii*,* Candida albicans*, and their single-species biofilms by agar dilution and broth microdilution test methods. Both agents inhibited the growth of all planktonic species. On the other hand, CHX exhibited lower minimum bactericidal concentrations than EEP against biofilms of* A. actinomycetemcomitans*,* S. aureus*, and* E. faecalis* whereas EEP yielded a better result against Lactobacilli and* P. intermedia*. The bactericidal and fungicidal concentrations of both agents were found to be equal against biofilms of Streptecocci,* P. gingivalis*,* A. israelii*, and* C. albicans*. The results of this study revealed that propolis was more effective in inhibiting Gram-positive bacteria than the Gram-negative bacteria in their planktonic state and it was suggested that EEP could be as effective as CHX on oral microorganisms in their biofilm state.

## 1. Introduction

Toothbrushing and interdental flossing are still basic methods to remove or to control bacterial plaque, which leads to the formation of caries and periodontal disease. However, the majority of the population may not perform the mechanical plaque removal sufficiently [1]. Thus, antimicrobial mouth rinses may provide an effective way of controlling bacterial plaque. It has been shown that chemotherapeutic mouth rinses are an effective adjunct to regular brushing and flossing [2].

Clinicians frequently administer CHX mouth rinses in order to inhibit the development of plaque [3, 4]. However, the cytotoxic characteristics [5] and side effects [6] of CHX are the basic disadvantages that limit the administration of this pharmaceutical. Some manufacturers are in an attempt to produce natural oral care products from plant extracts in order to avoid the side effects of synthetic products. Among these natural products, propolis comes forward due to its antimicrobial activity against a wide range of Gram-positive and Gram-negative pathogenic microorganisms [7–10]. Nevertheless, its effects against oral pathogens were compared to other oral antiseptics in a limited number of in vitro studies [11–13].

Propolis, also referred to as “bee glue,” is the generic name for the resinous substance collected from various plant sources by honeybees (*Apis mellifera*). In nature, honeybees use propolis for structural sealing of the hive. Although this product has gained acceptance in folk medicine for a thousand years, it has been recently rediscovered by researchers [14]. The chemical composition of propolis varies depending on regional, seasonal, and vegetational changes in plant sources from which it is collected by the bees [8, 15]. The application of propolis against a broad spectrum of oral bacteria may be beneficial for improving oral health. In addition, current opinion is that the use of standardized preparations of propolis is safe and less toxic than many other synthetic drugs [16–20]. The results of these studies also indicated that flavonoids were the primary biologically active constituents of propolis extracts.

This study was undertaken to compare the in vitro antimicrobial efficiencies of propolis and CHX against planktonic strains and their biofilm forms of ten different oral bacteria and a yeast-like fungus, which were commonly seen in oral microflora.

## 2. Material and Methods

In this study, 0.2% of CHX oral rinse solution (Drogsan, Turkey) and the ethanolic extract of propolis (EEP) were applied on the strains of* Streptococcus mutans* (*S. mutans*) ATCC#25175,* Streptococcus sobrinus* (*S. sobrinus*) ATCC#33478,* Lactobacillus acidophilus* (*L. acidophilus*) ATCC#4356,* Lactobacillus salivarius *subsp*. salivarius *(*L. salivarius *subsp*. salivarius*)* ATCC#11741, Enterococcus faecalis* (*E. faecalis*) ATCC#29212,* Staphylococcus aureus *(*S. aureus*) ATCC#25923,* Aggregatibacter actinomycetemcomitans* (*A. actinomycetemcomitans*) ATCC#29523,* Actinomyces israelii* (*A. israelii*) ATCC#12102,* Porphyromonas gingivalis *(*P. gingivalis*) ATCC#33277,* Prevotella intermedia* (*P. intermedia*) ATCC#25611, and one yeast-like fungus:* Candida albicans *(*C. albicans*) ATCC#10231. The minimum inhibitory concentration (MIC) and the minimum bactericidal concentration (MBC) for both antimicrobial agents were determined by conducting agar dilution and broth microdilution test methods.

### 2.1. Preparation of Propolis Extracts

The unrefined propolis (*Apis mellifera)* was obtained from Kazan/Ankara/Turkey. 20 g of unrefined propolis was accurately weighed (Shimadzu EB-330 EU, Japan) and dissolved in 100 mL of 80% ethanol (Sigma-Aldrich, USA) via ultrasonic bath at 40°C for 2 hours. The EEP solution was filtered through Whatman^®^ and Protran^®^ nitrocellulose membranes (Sigma-Aldrich, USA). The supernatant was evaporated by nitrogen flow until it dried. Approximately 5 *μ*g of residual substance was mixed with 75 *μ*L of dry pyridine and 50 *μ*L of bis-trimethylsilyl trifluoroacetamide (BSTFA), which was then heated at 80°C for 20 min. The final supernatant was analyzed by gas chromatography mass spectrometry (GC-MS).

### 2.2. GC-MS Analysis

Extracted propolis substrates were dissolved in ethanol and analyzed in a GC-MS device (17A QP5050 model, Shimadzu, Japan). The GC was operated in splitless injection mode by utilizing a DB-5 capillary column (length = 30 m, internal diameter = 0.2 mm, and film thickness = 0.1 mm) with an injection port temperature at 250°C. The carrier gas was helium at a flow rate of 50 mL/min. The column temperature was programmed so as to start from 60°C and increase up to 250°C in 15°C/min. increments. The initial and final time periods were 3 and 30 minutes, respectively. GC-MS was operated in full scan mode under 70 eV in an electron ionization mode of ionization energy. The ion source temperature was 230°C and the capillary direct interface was heated up to 230°C. The GC-MS peaks were identified by comparison to the data from Wiley 138 and Nist 98 libraries mentioned in the GC-MS program, which was supplied by the manufacturer. The chemical composition of EEP is given in Table 1.

### 2.3. Preparation of Microorganisms

*S. mutans* and* S. sobrinus* were cultured in 5 mL of brain heart infusion broth (BHIB, Oxoid, UK);* L. acidophilus* and* L. salivarius *subsp*. salivarius *were cultured in 5 mL of MRS Broth (DeMan Rogosa and Sharpe Medium, Merck, Germany) at 37°C for 48 hours in the microaerophilic atmosphere composed of 5% CO_2_.* E. faecalis* and* S. aureus* were aerobically cultured in 5 mL of brain heart infusion broth (BHIB, Oxoid, UK) at 37°C for 48 hours.* C. albicans* was cultured in an autoclaved-sterilized Sabouraud dextrose broth (SDB, Oxoid, UK) at 37°C for 48 hours under aerobic conditions.* A. actinomycetemcomitans*,* A. israelii, P. intermedia,* and* P. gingivalis* were cultured in autoclaved-sterilized fastidious anaerobe broth (Lab M, UK) supplemented by sheep blood (50 mL/L), vit. K (1 *μ*g/mL), and hemin (5 *μ*g/mL) at 37°C for 6-7 days in an anaerobic chamber (Electrotek, United Kingdom) in the atmosphere consisting of 90% N_2_, 5% CO_2_, and 5% H_2_. The last three ingredients were filter-sterilized by a 0.22 *μ*m millipore before adding to the main medium. All freshly grown bacterial suspensions in 5 mL of their specific broth media were suspended to 1.5 × 10^8^ CFU (colony forming unit)/mL according to the turbidity of 0.5 McFarland test standard, and the concentrations of the bacterial/fungal suspensions were adjusted spectrophotometrically by using an automatic Elisa reader (ELx800, Biotek, USA) at an optical density of 600 nm (OD_600_) to match the turbidity of all of the suspensions with 0.5 McFarland test standard.

### 2.4. Determination of the Minimum Inhibitory Concentration (MIC)

The agar dilution method was used to evaluate the inhibitory effects of EEP and CHX. The MIC values were determined according to the guidelines by Clinical Laboratory Standards Institute (CLSI) [21, 22]. Serial twofold dilutions of EEP and CHX solutions were prepared under aseptic conditions. The final concentrations of the dilutions of each agent, which ranged from 1024 to 0.5 micrograms/mL, were added to their specific agar media. Approximately 20 mL of the agar media was added to the sterilized plates (100 mm), which were prepared for each strain. All of the bacterial suspensions were cultured in their specific agar media plates and tested for the antimicrobial efficiencies of EEP and CHX dilutions.

*S. mutans* and* S. sobrinus* were incubated into trypticase soy agar (TSA, Difco, USA), which was supplemented with 20% sucrose (w/v), in the atmosphere composed of 5% CO_2_ at 37°C for 48 hours.* E. faecalis* and* S. aureus* were aerobically incubated into 5% sheep blood agar at 37°C for 48 hours.* L. acidophilus* and* L. salivarius *subsp*. salivarius* were incubated into MRS agar (Rogosa, Merck, Germany) in the atmosphere consisting of 10% CO_2_ at 37°C for 72 hours. Under aerobic conditions,* C. albicans* was incubated into Sabouraud dextrose agar (SDA, Oxoid, UK) at 37°C for 48 hours.* A. actinomycetemcomitans*,* A. israelii, P. intermedia,* and* P. gingivalis* were incubated into Colombia agar (Merck, Germany) (41.0 g/L), which was supplemented with sheep blood (50 milliliters/L), vit. K (1 *μ*g/mL), and hemin (5 *μ*g/mL), at 37°C in anaerobic chamber (90% N_2_, 5% CO_2_, and 5% H_2_) for 4-5 days. The MIC values for all of the test bacteria were defined as the lowest concentrations of EEP and CHX, which inhibited the visible growth of microorganisms. The agar plates, which did not contain EEP and CHX solutions and the ethanol solution of 80%, were used as controls.

By using the same EEP and CHX concentrations (ranging from 1024 to 0.5 *μ*g/mL), all of the test microorganisms were also analyzed by broth microdilution method. The purpose of this reanalysis was to corroborate the MIC results obtained by both methods and to find out the exact MBC/MFC values. 96-well microplates were used in the broth microdilution method. In this method, the dilutions of 0.1 mL of EEP and CHX, which were suspended in concentrations ranging from 1024 to 0.5 *μ*g/mL, were added to microplate wells. Subsequently, the 0.1 mL suspension of each microorganism in its specific broth media, which was prepared for the agar dilution method, was added to microplate wells containing 100 *μ*L of different concentrations of EEP and CHX. In order to determine the MIC values, the incubations were carried out in accordance with the same aerobic, microaerophilic, and anaerobic conditions and time intervals as mentioned before.

### 2.5. Determination of the Minimum Bactericidal Concentration (MBC) and the Minimum Fungicidal Concentration (MFC)

The MBC and MFC were determined by subculturing the 50 *μ*L of aliquots into their specific agar media. The aliquots were obtained from each microplate well in which no visible growth of microorganisms was noticed. The plates were incubated and cultured in the same conventional microbiological conditions and incubation periods as described before and were evaluated according to the guidelines of CLSI.

The 80% of ethanol was used as control against planktonic strains and the microbial biofilm of them. The results were expressed both in MIC and in MBC values. All tests were performed in duplicate.

### 2.6. Preparation of the Biofilms of the Test Microorganisms

Single-species biofilm of the microorganisms was generated on nitrocellulose membranes with 0.22 *μ*m pore size, 13 mm diameter (F7148, Sigma, USA), and then they were put on the flat-bottom 24-well tissue culture plates (3574, Corning Costar, USA). A volume of 100 *μ*L of each bacterial and fungal suspension, which were suspended according to the turbidity of 0.5 McFarland standard as a concentration of 1.5 × 10^8^ CFU/mL, was added to the surface of membranes and incubated for 30 min. Then 1 mL of their specific broth media as mentioned before was added to the wells. The plates were covered up and the microorganisms were cultured in their specific broth media and at the same atmospheric conditions as described before. The plates were put in the incubators with 5% CO_2_ for* Streptococci *and* Lactobacilli* and without 5% CO_2_ for* S. aureus*,* E. faecalis,* and* C. albicans* for 1 week. For* A. actinomycetemcomitans*,* P. gingivalis*,* A. israelii,* and* P. intermedia,* the culture plates were prepared in an automatic anaerobic chamber (Electrotek, UK) and incubated for at least 10 days in anaerobic conditions (10% H_2_, 10% CO_2_, and 80% N_2_) at 37°C. During their incubation period the broth media were meticulously changed with the fresh one in every two days avoiding touching the bottom of the wells. The tests were carried out in triplicate for each agent and microorganism. After incubation, the formation of biofilm was controlled by staining one of the membranes with 1% of safranin for each strain and evaluated microscopically. After the formation of biofilm layer, 1 mL solution of both test agents was put into the wells including the media at the concentrations of 1/2 diluted ranges as described and prepared before. The membranes were then discarded gently from the wells of the plates and were washed with PBS (pH: 7) 3 times. The membranes were then taken and put into other tubes including 1 mL PBS. Tubes were vortexed for 1 min. The suspensions inside the tubes were diluted 10^−4^, 10^−5^, and 10^−6^. Then, 100 *μ*L of each diluted suspension was put into plates including their specific agar media for each microorganism. After incubating at the same specific conditions for each strain the viable colonies of microorganisms were determined as MIC and MBC/MFC values.

## 3. Results

The EEP solution inhibited the growth of all planktonic species as much as CHX except* P. gingivalis* and* A. actinomycetemcomitans* (Table 2). The effect of EEP was not related to 80% of ethanol since it did not affect the growth of any bacteria and the fungus. In addition, EEP was more effective against Gram-positive bacteria and* C. albicans* than Gram-negative bacteria. Among the anaerobic bacteria, EEP seemed to be more effective on* P. intermedia* than CHX.

The analysis of the biofilms of tested microorganisms revealed that both test agents were not as effective as they were on planktonic species (Table 3). However, the concentration of both agents was found to be sufficient to manifest a bactericidal effect on tested bacteria and fungus except* S. mutans, S. sobrinus, and A. israelii*, which can be accepted as pioneer microorganisms in the formation of microbial dental plaque. The bactericidal effect of EEP was better than that of CHX on* L. acidophilus, L. salivarius *subsp*. salivarius,* and* P. intermedia*. The strongest effect of EEP was observed on* C. albicans and E. faecalis*. On the other hand, CHX was more bactericidal than EEP against* S. aureus*,* A. actinomycetemcomitans,* and* E. faecalis*.

## 4. Discussion

The results of this study indicated that both EEP and CHX had a range of inhibitory effects on the test species in their biofilm and planktonic state. In addition, this effect did not seem to be associated with the 80% ethanol in which the propolis was dissolved.

The antibacterial effects of propolis against microorganisms could be complex, leading to the disintegration of the cytoplasm, cytoplasmic membrane and cell wall, partial bacteriolysis, and inhibition of protein synthesis [23]. In a previous study, it was claimed that the pH and the concentration of propolis might alter due to solvents, and acidic propolis solutions were more effective on bacteria [24]. In addition, bacterial cell wall and their biofilm properties were concluded as adjunct factors, which determine bactericidal effect of propolis [25–27]. Thus, propolis could act against each microorganism in different ways.

The slight differences between our MIC/MBC values and those found in other studies are possibly owing to the differences in the strains and/or to the diverse origins of the propolis samples, since the composition of propolis depends on the regional vegetation [28]. The propolis samples obtained from poplar buds, which appear to be the dominant propolis source, in temperate zones (Asia, Europe, North America, etc.) predominantly contain phenolic compounds, including several flavonoids, aromatic acids, and their esters [29]. Mechanisms of activity of propolis against microorganisms are still not well understood. Some components present in propolis extracts like flavonoids (quercetin, galangin, and pinocembrin) and caffeic acid, benzoic acid, and cinnamic acid probably act on the microbial cytoplasmic membrane or cell wall site, causing functional and structural damage [28, 30]. Some authors revealed that its activity against microorganisms was more related to the synergistic effect of flavonoids (and other phenolics) than individual compounds [31]. Although our results indicated that the flavonoid content of the EEP utilized in this study was comparatively less than that reported by other studies [7, 32] on Turkish propolis, the cinnamic acid content was exceedingly high. This result could suggest a synergistic effect between cinnamic acid compounds and flavanoids.

Some authors stated that propolis could only be active against Gram-positive bacteria [33, 34] and some fungi [10] and still according to others it is less effective against Gram-negative bacteria [15, 17]. The MIC/MBC values determined in our study were in line with other studies stating that Gram-positive bacteria were more susceptible to propolis than the Gram-negative bacteria in their planktonic state. However, the results of the analysis of the biofilms did not corroborate the result indicating that propolis had been more effective on Gram-positive bacteria.

Previous reports elucidated that the biofilm of cariogenic bacteria was responsible for the formation of exopolysaccharides due to glucosyltransferase enzyme activity. Therefore, it was claimed that the aforementioned bacteria were highly tolerant to environmental stresses [25–27]. Certain studies reported that propolis led to a significant reduction in dental plaque [35] and prevented caries formation [18, 36], whereas others revealed that it had no significant effect on the reformation of dental plaque [37]. The results of this study revealed that propolis might be as effective as CHX on cariogenic bacteria. However, our results indicated that the current concentration of EEP might not be sufficient to show bactericidal effect on cariogenic bacteria. The inconsistency between the results of our study and those of others may be related to the factors above that indicate a correlation between the constituents of propolis, their activity on different bacterial cell wall structures, and the cellular activity of cariogenic bacteria.

The studies that investigated the effect of propolis on periodontal pathogens showed similar results to our study [8, 38, 39]. However, previous studies differ from ours because they aimed to investigate the effect of propolis on planktonic bacteria. The resistance of the biofilm of periodontopathogens selected for this study has been attributed to their extra polymeric substance (EPS) and lipopolysaccharide (LPS) levels. Similar cell wall structures and EPS of these periodontopathogens may explain why EEP has shown equal MIC/MBC values in their biofilms.

Biofilm formation of* C. albicans* is a critical issue in the treatment of* Candida *infections and can be a major challenge for clinicians. Biofilm resistance of* C. albicans* is not properly explained [40, 41]. It has been proffered that the biofilm resistance of propolis is not only correlated with EPS but develops over time [42] and related to the specific surface-induced gene expression [43]. In our study, in accordance with the results of a previous study, EEP displayed a strong antifungal activity against the* Candida *strain [33]. The strong activity of EEP on* C. albicans* may be related to its biofilm properties, which were stated in previous studies.

CHX, which is a gold standard, was selected as a test antiseptic for this study because of its wide-range effect on several microorganisms and its property known as “substantivity.” The MBC values observed for the bacteria and the fungus in their biofilm state indicated that there were no marked differences in the resistance of the biofilm of microorganisms against CHX or EPS. This result may suggest that the biofilm of tested microorganisms may respond in the same way to the current concentrations of both agents. CHX was apparently more effective on* E. faecalis. *Although this result was also corroborated by other studies, the studies were carried out on the planktonic form of* E. faecalis* [44, 45].

The main limitation of our study was the employment of MIC and MBC methods, which were performed by using broth microdilution and agar dilution techniques. While these methods are routine, unforeseen interactions between media constituents, with either one or more of the test agents, or the possible volatility of an important ingredient of the test mixture, such as alcohol, could prevent the interpretation of the results. Nonetheless, the MIC and MBC are the most reliable and easily interpreted methods for comparison of the formulations in use today [46].

## 5. Conclusion

Based on our results, we may conclude that the administration of propolis at appropriate concentrations might be effective on oral microorganisms. Although CHX is still one of the most common oral rinse products against wide range of microorganisms, EEP may serve as an alternative natural and reliable antimicrobial mouth rinse in order to avoid the side effects of CHX. In vivo studies are required to find out the effective mechanism of propolis and its appropriate administration dose on biofilm.

## Figures and Tables

**Table 1:** Chemical composition of EEP.

Chemical compound group	Chemical compound	%
Aromatic alcohols	Phenylethyl alcohol	0.15
	Z,Z-2,6-Dimethyl-3,5,7-octatriene-2-ol	0.12
	2-Naphthalenemethanol	0.8
	4-(1,1-Dimethylethyl)-benzenemethanol	0.05

Aromatic acids	Benzoic acid, ethyl ester	0.02
	2-Hydroxy-6-heptadec-8Z,11Z,14Z-trienylbenzoic acid	0.15

Aromatic heterocyclic alkaloid	6H-Benzofuro[3,2-c][1]benzopyran, 6a,11a-dihydro-3,4,8,9-tetramethoxy	0.11
	2-Trifluoromethyl-imidazole	1.90
	2-Trifluoromethyl-imidazole	0.11

Cinnamic acid and its esters	Hydrocinnamic acid, ethyl ester	0.03
	Cinnamic acid	0.04
	3-Methoxycinnamic acid	1.01
	Cinnamic acid, 3,4-dimethoxy methyl ester	0.14
	3,4-Dimethoxycinnamic acid	1.57
	Ferulic acid	0.08
	3-Hydroxy-4-methoxycinnamic acid	0.31
	m-Hydroxycinnamic acid	0.91

Flavanone	5,7-Dihydroxy-dihydroflavone	3.43
	Tectochrysin	2.80
	3,5,7-Trihydroxy-4′-methoxyflavone	0.08

Flavonones	Chrysin	12.06
	4′,5-Dihydroxy-7-methoxyflavanone	0.65
	4H-1-Benzopyran-4-one	5.8
	Pinostrobin chalcone	4.34
	Galangin	2.20

Linear hydrocarbons and their acids	Nonadecane	0.05
	2-Heptadecanone	0.05
	2-Nonadecanone	0.26
	Octadecane	1.69
	Nonadecane	0.92
	Z-14-Nonacosane	0.53
	9-Hexacosene	0.18

Naphthalene	1H-Cycloprop[e]azulene, decahydro-1,1,7-trimethyl-4-methylene	0.05
	3-Hydroxymyristic acid	0.16
	Myristinic acid	0.15
	cis-Oleic acid	0.97
	Palmitic acid	4.51
	Palmitic acid, ethyl ester	0.49
	Z-7-Tetradecenoic acid	0.11
	1,9-Tetradecadiene	0.12
	Oleic acid	3.17
	Ethyl oleate	2.05
	Stearic acid	0.52
	Linoleic acid	0.28

Unnatural amino acid derivatives	5-Aminovaleric acid	0.25
	5-Phenyl-4-pentenoic acid	1.31

**Table 2:** MIC and MBC/MFC values of EEP and CHX on the planktonic test microorganisms (*μ*g/mL).

Strains	Agar dilution	Broth microdilution	Agar dilution	Broth microdilution
	EEP (MIC/MBC)	EEP (MIC/MBC)	CHX (MIC/MBC)	CHX-MIC/MBC
*S*. *mutans*	4/8	4/8	8/16	16/16
*S*. *sobrinus*	8/8	4/8	8/8	8/16
*L*. *acidophilus*	4/4	4/8	8/8	4/8
*L*. *salivarius *subsp*. salivarius*	2/4	2/4	4/4	2/4
*A*. *actinomycetemcomitans*	64/128	64/128	32/16	16/32
*P*. *intermedia*	8/8	8/8	16/16	16/16
*P*. *gingivalis*	32/64	32/64	8/16	16/32
*S*. *aureus*	16/16	8/16	16/16	16/32
*E*. *faecalis*	8/8	4/8	8/8	8/16
*A*. *israelii*	16/16	8/16	16/16	8/16
*C*. *albicans*	16/16	8/16	16/16	16/32

**Table 3:** MIC and MBC/MFC values of EEP and CHX on the biofilms of the test microorganisms (*μ*g/mL).

Strains	EEP-MIC	EEP-MBC	CHX-MIC	CHX-MBC
*S*. *mutans*	1024	1024	1024	1024
*S*. *sobrinus*	1024	1024	1024	1024
*L*. *acidophilus*	512	512	512	1024
*L*. *salivarius *subsp. *salivarius*	512	512	512	1024
*A*. *actinomycetemcomitans*	128	256	64	128
*P*. *intermedia*	128	256	256	512
*P*. *gingivalis *	128	256	128	256
*S*. *aureus*	128	256	64	128
*E*. *faecalis*	64	128	16	32
*A*. *israelii*	512	1024	1024	1024
*C*. *albicans*	64	128	64	128
